# Visual trait variation and allometric scaling indicate intra- and inter-caste plasticity in the social wasp *Protopolybia sedula*

**DOI:** 10.1242/jeb.252873

**Published:** 2026-08-11

**Authors:** André Rodrigues de Souza, Priscila Araújo, Eduardo Fernando Santos, Fábio Santos do Nascimento, Amanda Prato, Wilson Franca, Emily Baird

**Affiliations:** ^1^ Departamento de Biologia Geral, Universidade Federal de Viçosa, 36570-900, Viçosa, Minas Gerais, Brazil; ^2^ Department of Zoology, Division of Functional Morphology, INSECT Lab, Stockholm University, 11418 Stockholm, Sweden; ^3^ Departamento de Ciências Biológicas, Instituto de Biociências, Letras e Ciências Exatas, Universidade Estadual Paulista “Júlio de Mesquita Filho”, São José do Rio Preto, 15054-000, Brazil; ^4^ Departamento de Biologia, Faculdade de Filosofia Ciências e Letras de Ribeirão Preto, Universidade de São Paulo, 14040-900, São Paulo, Brazil

**Keywords:** Caste specialisation, Eye plasticity, Allometry, Apposition compound eye, Vespidae

## Abstract

Understanding how phenotypic plasticity arises in sensory traits is a key challenge in evolutionary and developmental biology. Eusocial insects offer a useful model for exploring it, because their castes perform distinct tasks and occupy different light environments. We examined morphological variation and scaling in the visual systems of female castes of *Protopolybia sedula*, a neotropical wasp with simultaneous polygyny. Although queens are larger than workers, the two castes showed similar eye area, ommatidial diameter and ommatidia number. However, queens showed greater variability in ommatidial diameter, while workers varied more in eye area and ommatidia number. Allometric patterns also diverged: queen eye area scaled hypoallometrically and ommatidial diameter hyperallometrically with body size, whereas workers displayed isometric scaling for both traits. Analysis of ommatidia size and number indicates workers allocate more resources to increasing ommatidia number, while queens emphasise enlarging ommatidia. These differences highlight distinct developmental and scaling strategies that generate caste-specific visual adaptations.

## Teaser

**Summary:** Visual trait plasticity in the compound eyes of queens and workers of a social polygynous wasp may support task specialisation in workers and enhance social dominance advantages in queens.

## ADDITIONAL LANGUAGE ABSTRACT (PORTUGUESE)

Compreender como a plasticidade fenotípica em características sensoriais surge é um desafio fundamental na biologia evolutiva e do desenvolvimento. Insetos eussociais oferecem um modelo útil para explorá-la, pois suas castas desempenham tarefas distintas e ocupam diferentes ambientes luminosos. Examinamos a variação morfológica e a escala nos sistemas visuais de castas femininas de *Protopolybia sedula*, uma vespa neotropical com poliginia simultânea. Embora as rainhas sejam maiores que as operárias, ambas as castas apresentaram área do olho, diâmetro dos omatídios e número de omatídios semelhantes. No entanto, as rainhas mostraram maior variabilidade no diâmetro dos omatídios, enquanto as operárias variaram mais na área do olho e no número de omatídios. Os padrões alométricos também divergiram: a área dos olhos das rainhas apresentou escala hipoalométrica e o diâmetro dos omatídios hiperalométrica com o tamanho do corpo, enquanto as operárias exibiram escala isométrica para ambas as características. A análise do tamanho e do número de omatídios indica que as operárias alocam mais recursos para aumentar o número de omatídios, enquanto as rainhas priorizam o aumento do tamanho dos omatídios. Essas diferenças destacam estratégias distintas de desenvolvimento e escalonamento que geram adaptações visuais específicas para cada casta.

## INTRODUCTION

Phenotypic plasticity in sensory organs enables animals to exploit different ecological niches by adjusting sensitivity to, for example, varying light environments and cues, potentially promoting the evolution of specialised behaviours. Eusocial Hymenoptera provide an exceptional model for studying such plasticity because individuals share a highly similar genetic background and, in most species, caste differentiation occurs post-fertilisation (Wheeler, 1986). Despite this shared genetic framework, female castes exhibit pronounced morphological differences within and between castes that allow task specialisation (Oster and Wilson, 1978). These differences in behavioural roles are often accompanied by differential investment during development. Morphological castes are ultimately determined at the pupal stage, when individuals have finite resources to invest in different body structures during metamorphosis (Wheeler, 1991), potentially intensifying allocation constraints. Castes often differ in energetic priorities, with reproductive females (future queens) generally allocating more resources to their ovaries (Boggs, 1981; Llandres et al., 2015), as well as to energy storage (e.g. in the form of lipids) (Judd et al., 2010), often causing them to have larger gasters than workers (Strohm and Linsenmair, 1997; Smith et al., 2008). This increased investment in reproduction and storage may constrain allocation to other costly systems, such as sensory organs, potentially contributing to caste-specific differences in sensory organ design.

As eyes are among the most energetically costly organs to build and maintain (Niven and Laughlin, 2008), insect visual systems provide a powerful framework for investigating how potential trade-offs between reproduction and sensory investment shape phenotypic plasticity both between and within castes. Insect visual traits vary in response to both the light environment and behavioural ecology (Kittelmann and McGregor, 2024). In the case of insects with apposition compound eyes – such as Hymenoptera – variation in visual traits occurs through modifications to the size, distribution and number of structural units, called ommatidia (Kapustjanskij et al., 2007; Streinzer et al., 2013; Perl and Niven, 2016; Narendra et al., 2016; Taylor et al., 2019; Araújo et al., 2023). For example, honeybee queens possess fewer ommatidia than workers despite having similar eye sizes (Streinzer et al., 2013), a pattern consistent with differences in investment associated with reproductive roles and reduced reliance on vision compared with workers, which perform visually demanding tasks such as foraging and nest-site selection. Within castes, visual traits can also vary with body size: in *Bombus terrestris*, larger workers have larger eyes and facets (Taylor et al., 2019; Kapustjanskij et al., 2007), enabling them to forage in dim light, whereas smaller workers typically remain inside the nest where vision is less critical (Kapustjanskij et al., 2007; Garófalo, 1978).

Allometric analyses, which describe how the size of a body part scales with body size, provide a useful tool for investigating the developmental basis for phenotypic plasticity in visual traits. In the ant *Formica rufa*, allometric analyses revealed that small workers invest proportionally more energy in visual structures than large workers (Perl and Niven, 2016). Variation in allometric slopes for different sensory traits has also been described at the colony level in ants and bumblebees, indicating that the developmental rules that dictate how sensory traits scale with body size are plastic (Perl and Niven, 2016; Perl et al., 2022). Although these studies reveal the value of applying allometric analyses to eusocial Hymenoptera to understand the drivers of phenotypic plasticity in sensory traits, direct caste comparisons remain rare, with existing analyses relying on small queen sample sizes (Streinzer et al., 2013; Narendra and Ribi, 2017). Furthermore, all published work has focused on species with monogynous colonies [with the exception of the study by Perl and Niven (2016), but they analysed only workers], and no study has examined how visual traits scale in polygynous species, where many queens co-occur. This gap is notable because polygyny may promote greater phenotypic diversification among queens. Consequently, key questions remain unanswered. Do coexisting queens and workers differ in their visual traits? If so, are these differences explained by body size alone, or do they reflect additional variation associated with caste-specific investment? Furthermore, do queens and workers exhibit distinct allometric relationships in visual traits that are consistent with differences in their primary tasks?

To address these questions, we studied *Protopolybia sedula* (Hymenoptera: Vespidae), a small neotropical swarm-founding wasp with simultaneous polygyny (Chavarría Pizarro et al., 2023) (Fig. S1). We compared body size and compound eye morphology between queens and workers, quantified within-caste variation in visual traits as a proxy for phenotypic plasticity, and used allometric analyses to test whether queens and workers differ in their investment in visual structures relative to body size. We interpret these patterns in the context of *P. sedula's* natural history, behavioural roles and caste-specific light environments.

## MATERIALS AND METHODS

### Study species and sample collection

Colonies of *Protopolybia sedula* (de Saussure 1854) are active throughout the year, and simultaneous polygyny is common, making it possible to sample many queens and workers from the same colony. Workers forage for prey and nest-building materials, defend the colony and participate in swarming (Richter, 2000; Detoni and Prezoto, 2021), activities that depend heavily on vision. In contrast, queens remain inside the shaded nest (West-Eberhard, 1978; Herman et al., 2000; Noll et al., 2021), laying eggs and leaving only to swarm, or mate once in their lifetime (Strassmann, 2001; Detoni and Prezoto, 2021).

Sampling was conducted between January and June 2022 in gardens and buildings in Ribeirão Preto, São Paulo State, Brazil. Each colony was collected with a bag at night, around 20:00 h. At this time, foraging activity has ceased, and nearly all wasps are on the nest, thus avoiding sampling bias. A total of 175 wasps (87 queens and 88 workers) from five colonies were collected. Upon collection, wasps were immediately killed by cooling at −24°C, after which their caste was determined and their morphological traits were measured.

### Caste assignment

A stereomicroscope was used to examine individuals and assign caste. The first screening was based on gaster size, which is slightly larger in queens (A.R.d.S., personal observation). Caste was then confirmed following Prato et al. (2021). Individuals were assigned as queens if they had activated ovaries, mature oocytes, sperm in the spermatheca and no evidence of wing wear upon visual inspection (indicating little flight activity and therefore unlikely to be workers). Females with inactivated (filamentous) ovaries with no visible oocytes, no sperm in the spermatheca and some degree of wing wear (indicating regular flight activity) were assigned as workers.

### Trait measurements

The inter-tegular distance, commonly used as a proxy for body size in Hymenoptera (Cane, 1987), was measured using a stereomicroscope equipped with a scaled lens and image capture system. Compound eye replicas were created by applying nail polish to the left eye surface to form a mould that, after 24 h, was carefully removed, flattened between glass slides and immediately photographed (Ribi et al., 1989) (Figs S1 and S2). From each replica, we measured eye area, ommatidial diameter and ommatidia number.

Eye area, ommatidial diameter and ommatidia number were measured using ImageJ. Eye area was quantified by tracing the outer contour of the compound eye using the *Polygon selection* tool. To estimate mean ommatidial diameter, a grid of equal-sized rectangles was overlaid on each eye image (Fig. S2). Within each grid cell, ommatidial diameter was estimated by drawing a straight-line transect through the centre of 10 adjacent ommatidia aligned in a regular ommatidial row using the *Straight Line* tool in ImageJ. The transect length was then divided by the number of ommatidia measured to obtain the mean ommatidial diameter. Because eye size varied among individuals, the number of grid rectangles used differed accordingly (minimum 20, maximum 34). The total number of ommatidia was counted manually for each eye using the *Multi-Point tool* in ImageJ.

### Statistical analysis

Rosner's tests were applied to detect and remove outliers in all measured traits (*n*=7 outliers, all for ommatidial diameter in workers). To test caste differences in body size, a type III ANOVA with Satterthwaite's method (Kuznetsova et al., 2017) was applied. For this, a linear mixed model was performed, including body size as the dependent variable and caste as the independent variable. Type III ANOVA with Satterthwaite's method was also applied to test caste differences in visual traits (eye area, ommatidia number or ommatidial diameter), which were included as dependent variables, while caste was used as an independent variable. Considering the difference between the queen and worker body sizes (result of type III ANOVA for body size), the interaction between caste and body size was included as an independent variable in order to eliminate any differential effect on body growth between castes. In this way, all type III ANOVA were used solely to verify whether queens and workers differed in visual trait measurements, while excluding possible effects of body growth on these traits. All variables were log transformed, and wasp nest origin was included as a random factor in all type III ANOVA.

Coefficients of variation (CV=standard deviation/mean×100) (Sokal and Rohlf, 1995) were used to quantify variation in visual traits within each caste to provide a proxy for phenotypic plasticity. Differences in CVs between queens and workers were tested using Fligner–Killeen tests for non-normal data (Fligner and Killeen, 1976) and Levene's tests for normally distributed variables (Fox and Weisberg, 2018).

To determine whether queens and workers share the same scaling relationships between eye traits and body size, we estimated standardised major axis (SMA) regressions (Warton et al., 2006). The SMA is a model II regression, which should be used when both the dependent and independent variables are random and have associated measurement error (Legendre and Legendre, 2012), such as in biometric studies. SMA regressions analyse allometric growth using the power function *y*=*a*·*xb* [where *y* is the visual trait, *x* is the body size, *a* is the elevation (intercept value) and *b* is the scaling exponent (slope value); Huxley and Teissier, 1936]. The SMA tests whether the scaling exponent indicates isometry (scaling exponent=1), hypoallometry (scaling exponent<1) or hyperallometry (scaling exponent>1), and whether it varies between castes (growth ratio), as proposed by Warton et al. (2006). An isometric relationship implies that the relative size of an organ remains constant as body size increases. Hypoallometry indicates that the organ becomes relatively smaller with increasing body size, whereas hyperallometry means the organ becomes relatively larger. All measurements were log-transformed for the SMA estimates.

Finally, to examine whether caste-specific allometric relationships could generate distinct eye designs associated with caste-specific tasks, we estimated SMA regressions between ommatidial diameter and ommatidia number. Under the cell size model (Chown et al., 2007), ommatidial diameter serves as a proxy for cell size, whereas ommatidia number reflects the number of cells contributing to eye growth. Thus, the scaling between these two traits reveals whether morphological variation arises primarily through changes in ommatidial size, number or a combination of the two. This approach captures the developmental trade-offs between enlarging individual ommatidia and increasing their total number (Chown et al., 2007), allowing us to infer whether queens and workers invest differentially in sensitivity (larger ommatidial size) or acuity (larger ommatidial number).

All statistical analyses were performed within the R environment (https://www.r-project.org/) using functions from the packages EnvStats (Millard, 2013), lmerTest (Kuznetsova et al., 2017), DHARMa (https://CRAN.R-project.org/package=DHARMa) and smatr (Warton et al., 2012). The significance level was set at *P*<0.05. All the dataset details can be checked in Table S2.

## RESULTS AND DISCUSSION

### Visual plasticity within and between castes

**Fig. 1.**
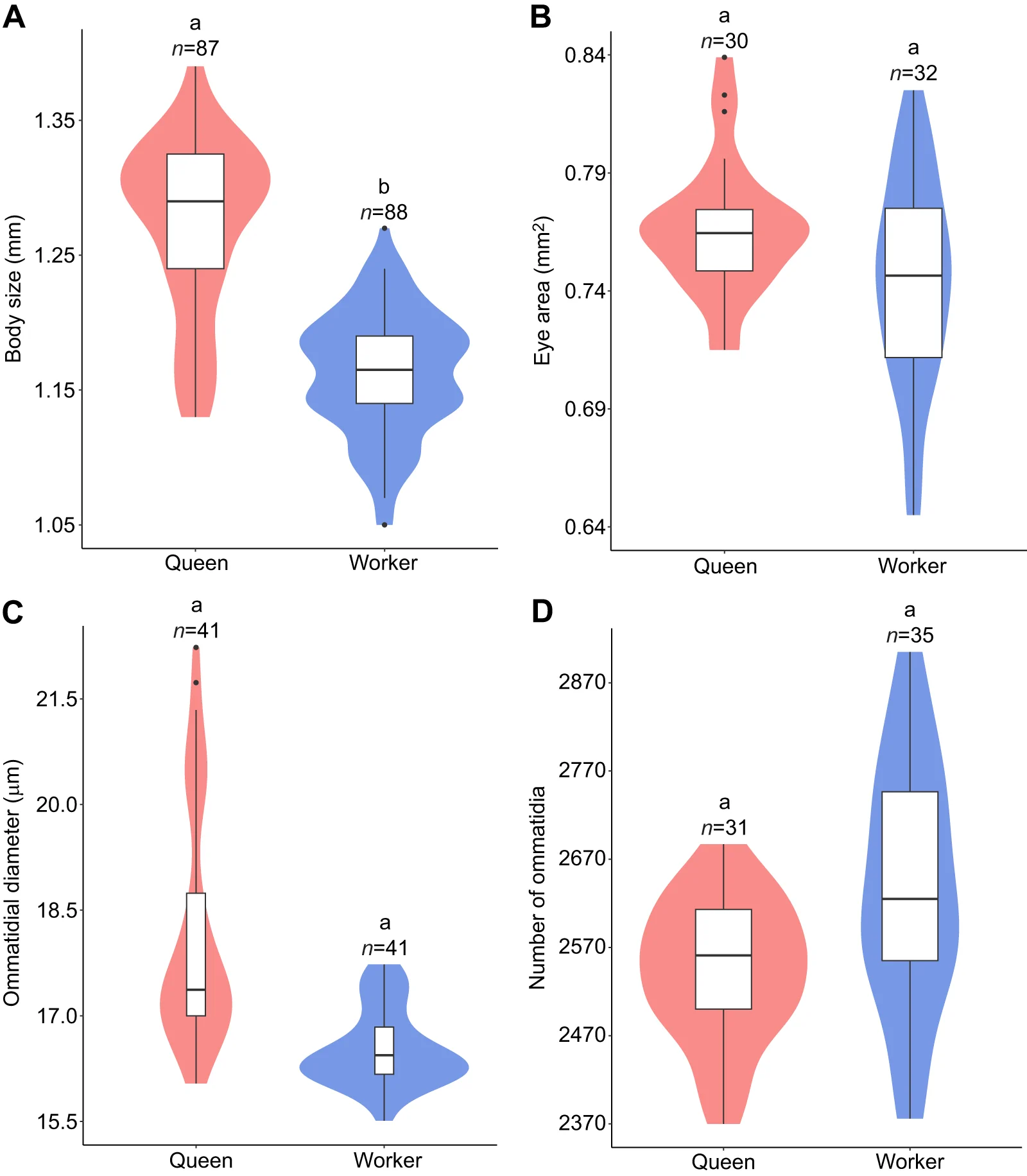
**Body size and visual traits of queen and worker *Protopolybia sedula* wasps.** Boxplots and violin plots show the morphometric differences in body size (A), eye area (B), ommatidial diameter (C) and ommatidial number (D) between queen (red) and worker (blue) castes. Different letters above the violin plots indicate statistical differences between castes (*P*<0.05) using type III ANOVA with Satterthwaite's method. Numbers (*n*) above the violin plots indicate sample size. Box plots indicate median (line), upper and lower quartiles (box limits) and 1.5× the interquartile range (whiskers).

Our analysis showed that queens of *P. sedula* are larger than workers (*F*_1_=242, *P*<0.001; Fig. 1A; Table S1). Despite this, queens and workers did not differ significantly in eye area, ommatidial diameter or the number of ommatidia (*F*_1_=0.4, *P*=0.53; *F*_1_=1.9, *P*=0.16; *F*_1_=3.9, *P*=0.052; respectively; Fig. 1B–D; Table S1). This pattern contrasts with that reported for some other social Hymenoptera, suggesting that the relationship between caste and eye morphology varies among taxa. In the stingless bee *Scaptotrigona postica*, the compound eye is slightly smaller in queens than in workers, with queens having a smaller ommatidial diameter but similar numbers of ommatidia (Ribi et al., 1989). Furthermore, honeybee queens possess eyes of similar size but with fewer ommatidia than workers despite substantial differences in body size between the castes (Streinzer et al., 2013). In contrast, bumblebee queens are substantially larger than workers (Röseler and van Honk, 1990) and have correspondingly larger eyes with more facets and larger facet diameters (Tichit et al., 2022).

Interestingly, some visual traits had a higher degree of variation than others: the variation for ommatidial diameter was almost three times higher in queens than in workers (CV=9.1% and 3.2%, respectively, χ^2^_1_=7.6, *P*=0.005). In contrast, the variation for eye area and number of ommatidia was statistically higher in workers than in queens (workers: CV=5.9% and 5.2%; queens: CV=3.6% and 3.0%; *F*_1_=5.7, *P*=0.02; χ^2^_1_=7.7, *P*=0.005; respectively). The variation for body size was similar between workers and queens (CV=3.9% and 5.0%, respectively; χ^2^_1_=2.6, *P*=0.1). The substantial variation in visual traits we observed in both queens and workers suggests that *P. sedula* exhibits considerable visual trait plasticity. Such plasticity may enable individuals within the same caste to optimise vision to different light environments. This variability could promote task specialisation, as suggested for bumblebees (Garófalo, 1978; Taylor et al., 2019): workers with poor eye resolution (i.e. smaller eye area and lower number of ommatidia) tend to perform non-visual tasks inside the nest, while workers with better eye resolution (i.e. larger eye area and higher number of ommatidia) may be more likely to perform outdoor tasks, where vision is essential for flight control, prey detection and landmark recognition around the nest site (Collett, 1995; Júnior et al., 2008). For queens, ommatidial diameter was the only visual trait with high variability, which may indicate differences in optical sensitivity among individuals, although alternative mechanisms, such as adjustments in focal length, cannot be ruled out.

### Queens and workers exhibit distinct allometry in visual traits

**Fig. 2.**
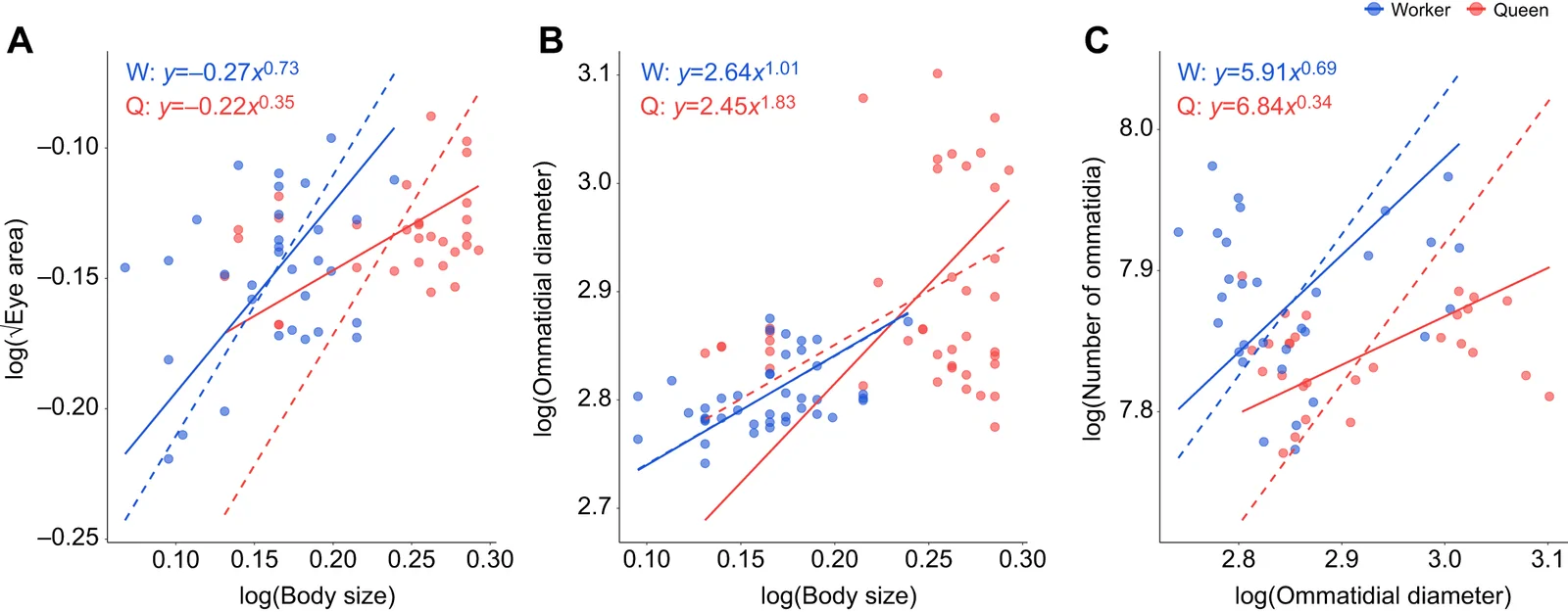
**Allometric analyses of visual traits in *P. sedula* queens and workers.** Allometric relationship (solid lines) of eye area (mm^2^; A) and average ommatidial diameter (μm; B) versus body size (mm), and number of ommatidia versus ommatidial diameter (C) in queens (red) and workers (blue). Dashed lines represent the theoretical isometric relationships for each caste (null hypothesis, H_0_). The equations show allometric growth for each caste: queen (Q: red) and worker (W: blue). Queen and worker sample sizes, respectively: (A) 30 and 32; (B) 41 and 41; (C) 28 and 31.

Although eye area and ommatidial diameter did not differ significantly between castes, the SMA analysis indicated that queens and workers follow different allometric patterns (Fig. 2A,B, Table 1). Eye area was isometric in workers (scaling exponent=0.73) – the relative investment in eye size remained constant across body size – but was hypoallometric in queens (scaling exponent=0.35), indicating that larger queens have relatively smaller eyes than smaller queens. As with eye size, worker ommatidial diameter scaled isometrically (scaling exponent=1.0) but was hyperallometric in queens (scaling exponent=1.83), indicating that larger queens had relatively larger ommatidia than smaller queens. These findings suggest that *P. sedula* queens and workers have distinct morphological castes that follow different developmental programmes. These developmental programmes determine not only whether the ovaries will develop and mature (Chavarría-Pizarro et al., 2023) but also how investment in visual traits varies between castes.

**Table 1.** Comparison of allometric slopes between and within castes

Explanatory variable	Response variable	Slope (95% CI)	H_0_: caste slopes are equal		H_0_: isometric slope	
			Likelihood ratio	*P*-value	Likelihood ratio	*P*-value
Body size	Eye area	Q: 0.349 (0.249–0.502) W: 0.730 (0.519–1.027)	8.393	0.004	Q: −0.796 W: −0.324	Q: <0.001 W: 0.07
	Ommatidial diameter	Q: 1.828 (1.338–2.498) W: 1.012 (0.758–1.350)	7.434	0.006	Q: 0.547 W: 0.0133	Q: <0.001 W: 0.934
Ommatidial diameter	Number of ommatidia	Q: 0.344 (0.234–0.504) W: 0.690 (0.477–1.000)	6.598	0.01	Q: −0.796 W: −0.355	Q: <0.001 W: 0.049

CI, confidence interval; H_0_, null hypothesis; Q, queen; W, worker.

The isometric scaling of eye size and ommatidial diameter in workers suggests that small workers have proportionally smaller eye areas and ommatidial diameters than larger workers. This reinforces the idea that workers differ in visual sensitivity and acuity, with smaller workers probably having poorer vision than larger ones (as has been shown for bumblebee workers; Kapustjanskij et al., 2007). However, these findings raise the question of why queens exhibit a different pattern of allometry – specifically, hypoallometry between eye area and body size, and hyperallometry between ommatidial diameter and body size. While hypoallometry is the most common scaling of morphological traits within species, hyperallometry is less common (Voje, 2016). Hyperallometric patterns are often associated with traits under strong selection related to competitive or reproductive performance, as theoretical models have shown that such selection pressures can drive disproportionate trait investment (Bonduriansky and Day, 2003; Voje, 2016). We suggest that the distinct allometric patterns in queen eye traits, together with the high variation in ommatidial diameter (see Figs 2B and 1C, Table 1), may facilitate vision-dependent behaviours that enhance reproductive success or dominance in colonies with multiple queens. In particular, queens with disproportionately larger ommatidia relative to body size probably have higher light sensitivity, which could improve their ability to detect or respond to key social or reproductive cues within the colony. These behaviours include the ‘bending display’ that queens use to measure the reproductive capacity of potential queens and the ‘worker dance’, which is a behaviour performed by workers that need to be recognised by queens to avoid being expelled from the hive, or to recognise and remove another queen from a brood cell because she has laid an egg, fixed an egg or performed cell inspection (West-Eberhard, 1978; Chavarría-Pizarro et al., 2018, 2023). Such visual enhancements may thus provide certain queens with a reproductive or social dominance advantage.

### Eye design reflects caste-specific behaviour

We found that ommatidia number scaled hypoallometrically with ommatidial diameter in both queens and workers (Fig. 2C, Table 1; scaling exponents=0.34 and 0.69, respectively). Thus, in both castes, individuals with larger ommatidia had proportionally fewer ommatidia than those with smaller ommatidia, reflecting a developmental trade-off between enlarging individual ommatidia and producing additional ommatidia. However, the scaling relationship differed significantly between castes (Fig. 2C, Table 1), indicating distinct caste-specific investment patterns in ommatidial units. The steeper slope in workers means that, relative to queens, they allocate more energy to producing a greater number of ommatidia per eye area – an energetically costly process that begins in the final larval instar (Miyazaki et al., 2010; Pichaud and Casares, 2022) and requires substantial resources to develop and maintain (Laughlin et al., 1998; Niven and Laughlin, 2008). In contrast, queens invest less in producing additional ommatidia and instead rely more heavily on enlarging them, consistent with the observation that queens possess the largest ommatidial diameters recorded in our dataset (Fig. S3), probably reflecting both their reduced need for high visual acuity in the dim nest environment and the competing energetic demands of developing ovaries with mature oocytes toward the end of larval and pupal development (Llandres et al., 2015). Thus, our analysis reveals that queen and worker eyes differ not only in their scaling exponents but also in the underlying developmental mechanisms – ommatidia size versus ommatidia number – that generate their caste-specific visual designs.

### Conclusions

Our results demonstrate that queens and workers of *P. sedula* exhibit substantial variation in visual traits and that part of this variation is explained by body-size scaling. Differences in caste-specific allometric relationships indicate that queens and workers follow distinct developmental trajectories, generating variation in eye morphology within each caste. Analyses of ommatidial size versus number revealed contrasting investment strategies: workers invest proportionally more in increasing ommatidial number, potentially enhancing visual acuity, whereas queens invest more heavily in enlarging ommatidia, potentially enhancing visual sensitivity. These differences are consistent with the distinct behavioural roles of the two castes. Workers perform both intranidal and extranidal activities, including tasks that rely heavily on vision, such as foraging and nest defence, whereas queens are primarily involved in reproductive and social interactions within the nest. In addition, the high variation in ommatidial diameter observed among queens may be relevant to behaviours associated with reproductive assessment and competition between them, although whether visual morphology influences reproductive success or social dominance remains unknown. Together, our findings show that phenotypic plasticity, caste-specific allometry and contrasting investment in ommatidial size versus number generate distinct visual phenotypes in queens and workers, providing a foundation for future studies investigating how variation in visual design relates to behavioural specialisation and reproductive interactions in swarm-founding social wasps.

## Supplementary Material

10.1242/jexbio.252873_sup1 Supplementary information

Table S2. Dataset. Excel file containing all morphometric measurements for *Protopolybia sedula* queens and workers, organized by nest and individual.
