# Supplementary material for: Visual trait variation and allometric scaling indicate intra- and inter-caste plasticity in the social wasp Protopolybia sedula
Source: J Exp Biol. 2026 Aug 11;229(15):jeb252873. doi: 10.1242/jeb.252873 (PMC13535097; doi:10.1242/jeb.252873)
Supplement: Supplementary file 1 [file jexbio-229-252873-s1.pdf]

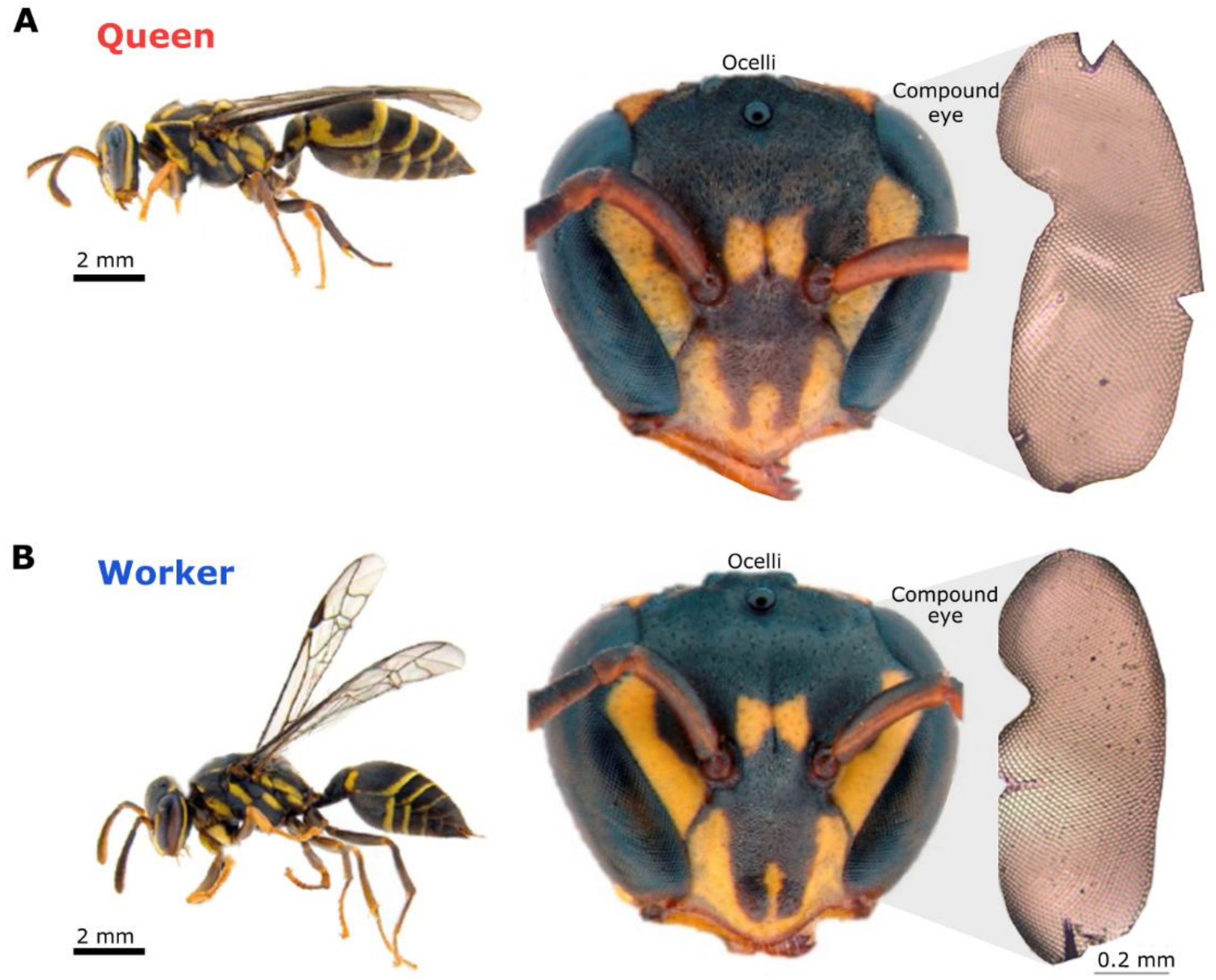

Fig. S1. Morphological comparison of *Protopolybia sedula* workers and queens. Overall appearance, eye mould, and head of a queen (A) and a worker (B). The heads are not scaled.

Journal of Experimental Biology • Supplementary information

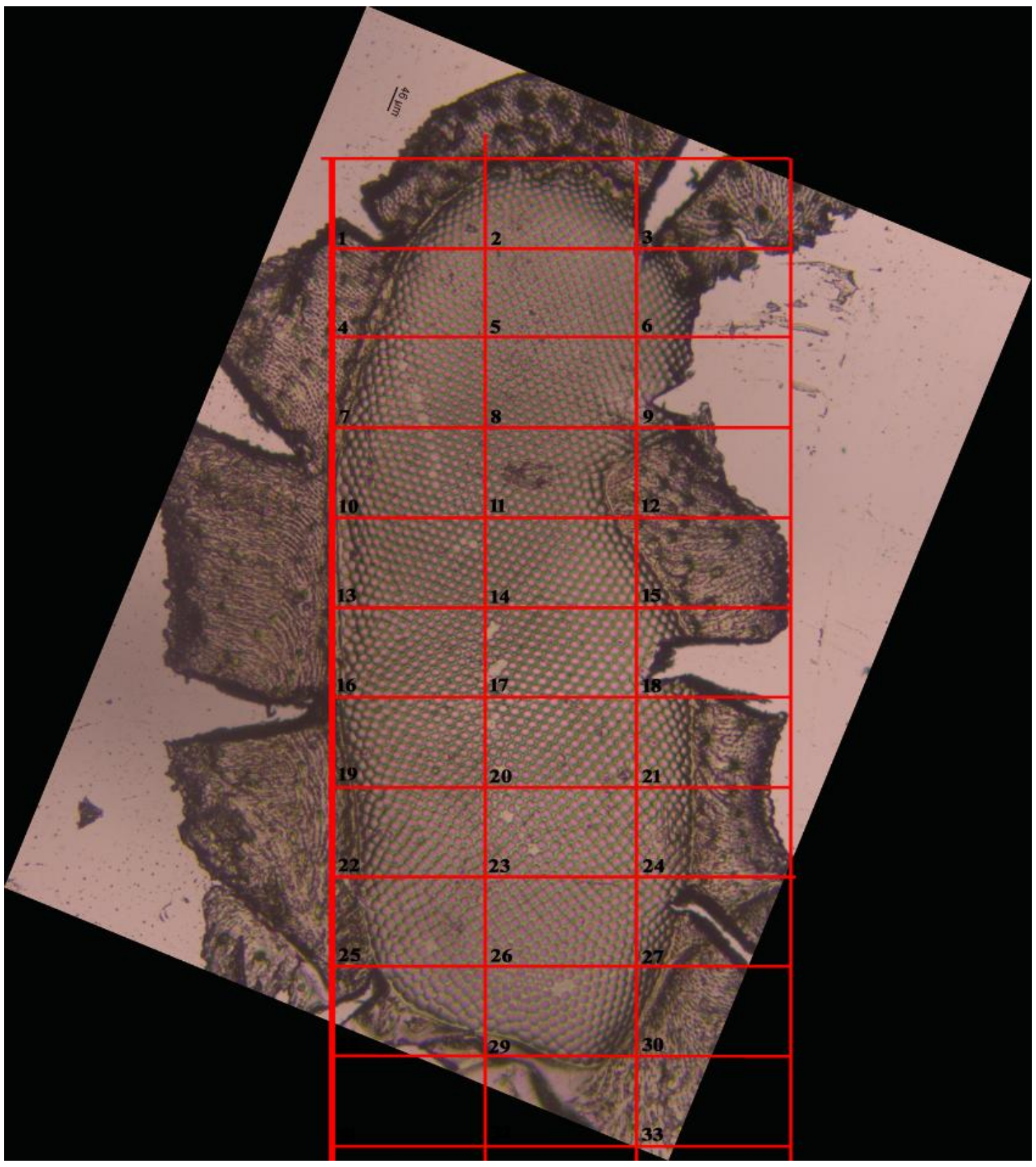

Fig. S2. Eye replica with a grid of equal-sized rectangles overlapped. Ommatidia diameter was measured in all rectangles where it was possible to measure 10 ommatidia aligned in a regular ommatidial row.

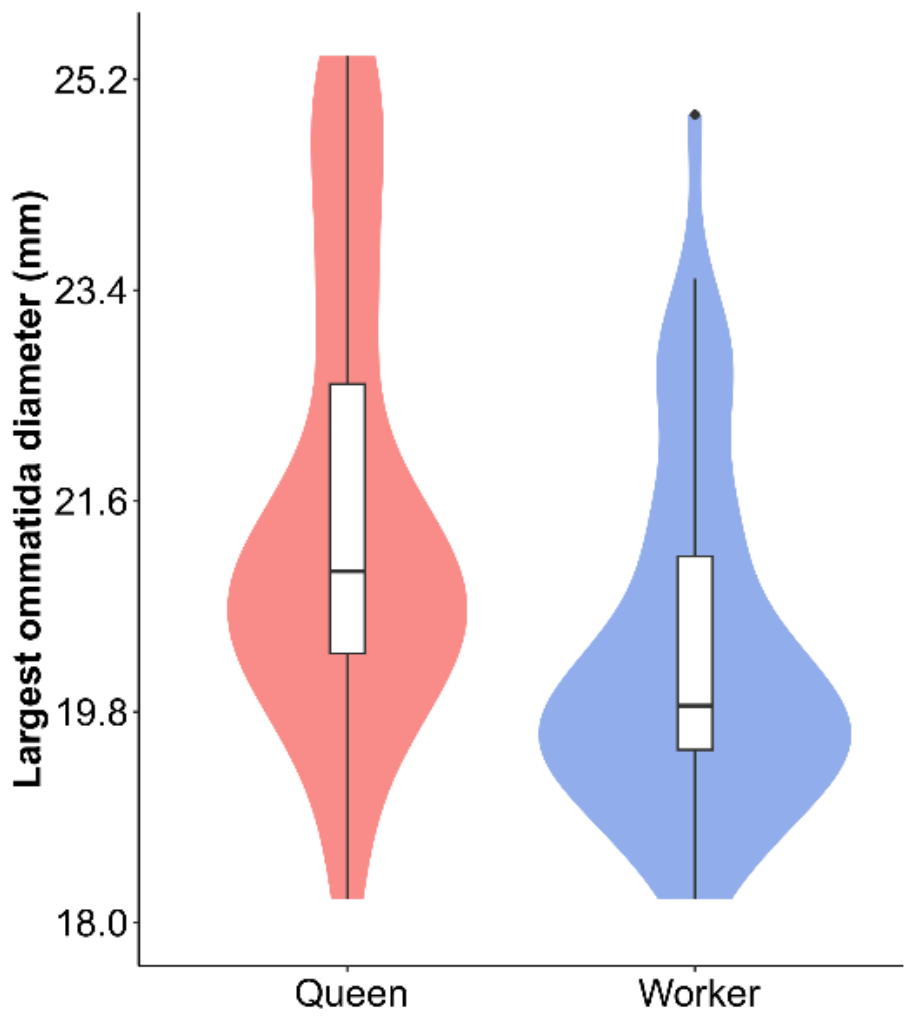

Fig. S3. Comparison of maximum ommatidial diameter between queens and workers. Queens exhibited larger maximum ommatidial diameters than workers (F = 4.4, df = 1, p = 0.03).

Table S1. Morphometric differences across castes. SEM= standard error of the mean, n = number of individuals.

Morphometric parameter	Queen (mean ± SEM (N))	Worker (mean ± SEM (N))	Significance tests (Type III ANOVA)	
			Statistics	p-value
Body size (mm)	1.27 ± 6.86e^{-03} (87)	1.16 ± 4.77e^{-03} (88)	$F_{1,169.030}$ = 242.090	<0.001*
Eye area (mm^2)	0.766 ± 5.09e^{-03} (30)	0.744 ± 7.75e^{-03} (32)	$F_{caste\ 1,58.634}$ = 0.401	0.529
			$F_{caste:body\ 1,58.030}$ = 5.829	0.005*
Ommatidia diameter (µm)	18.14 ± 0.007 (41)	16.52 ± 0.007 (41)	$F_{caste\ 1,75.106}$ = 1.986	0.163
			$F_{caste:body\ 1,75.144}$ = 9.466	<0.001*
Number of ommatidia	2433.55 ± 14.12 (31)	2638.20 ± 23.17 (35)	$F_{caste\ 1,62.951}$ = 3.936	0.052
			$F_{caste:body\ 1,62.864}$ = 3.104	0.052

Table S2. Dataset. Excel file containing all morphometric measurements for *Protopolybia sedula* queens and workers, organized by nest and individual.

Available for download at
https://journals.biologists.com/jeb/article-lookup/doi/10.1242/jeb.252873#supplementary-data

Journal of Experimental Biology • Supplementary information
